# Influence of living settings on physical activity levels and volition in exercise in male and female university students

**DOI:** 10.1371/journal.pone.0304579

**Published:** 2024-07-18

**Authors:** Federico Quinzi, Loretta Francesca Cosco, Francesca Greco, Katia Folino, Claudia Cerulli, Francesco Pio Oranges, Alessio Facchin, Maria Grazia Tarsitano, Gian Pietro Emerenziani

**Affiliations:** 1 Department of Experimental and Clinical Medicine, University “Magna Græcia” of Catanzaro, Catanzaro, Italy; 2 Department of Movement Sciences and Wellbeing, University Parthenope of Naples, Naples, Italy; 3 Department of Movement, Human and Health Sciences, University “Foro Italico” of Rome, Rome, Italy; 4 Department of Medical and Surgical Sciences, University Magna Græcia of Catanzaro, Catanzaro, Italy; Ovidius University of Constanta: Universitatea Ovidius din Constanta, ROMANIA

## Abstract

This study explored the influence of living settings on physical activity (PA) levels and volition in exercise and their correlation, considering sex differences. Five hundred and sixty-six university students (261 rural and 305 urban) from Calabria region (Italy) attending universities courses filled an online survey where Global Physical Activity Questionnaire and Volition in Exercise Questionnaire were administered. Rural females (RF) showed lower PA levels and self-confidence than rural males (RM) (p<0.01). Postponing training and unrelated thoughts were higher in RF than RM (p<0.01 and p<0.05 respectively). PA levels, self-confidence and coping with failure were lower in urban females (UF) than urban males (UM) (p<0.01), Postponing training and unrelated thoughts were higher in UF than UM (p<0.01 and p<0.05). In RF and UF, PA levels positively correlated with self-confidence (Rho = 0.397) and coping with failure (Rho = 0.330), whereas negatively correlated with postponing training (Rho = -0.487) and unrelated thoughts (Rho = -0.283). In RM and UM, PA levels positively correlated with self-confidence (Rho = 0.270) and coping with failure (Rho = 0.258), whereas it negatively correlated with postponing training (Rho = -0.285). PA levels positively correlated with reasons (Rho = 0.260) only in UM. We showed for the first time the relationship between PA and volition factors considering the living setting in university students. Sex differences were observed in some volition facilitators and PA levels independently by the living context.

## Introduction

The combination of physical and social factors in which actions unfold, also known as, the “behavior setting” concept enables us to understand how the environment influences physical activity participation. This concept, firstly introduced by Barker [1], offers insights into how different settings (e.g., homes, gyms, streets, parks, and workplaces) might impact the practice of physical activity.

Considering these setting-specific features may further our understanding of physical activity and its relation within different environmental contexts [2, 3]. Moreover, the place of birth could influence the development of an individual’s skills and physical activity participation [4, 5]. Indeed, it has been shown that different living settings might influence the physical activity partecipation due to the peculiar social and cultural characteristics [5–10]. Therefore, it is crucial to highlight the importance of the context in which individuals grew up and how it could influence participation in physical activity. In addition, interventions aimed at promoting physical activity may benefit from this information to adapt public health policies to specific contexts [3].

Mumu and colleagues [11] showed that rural residents reported significantly higher levels of moderate physical activity, as measured by the Global Physical Activity Questionnaire (GPAQ), compared to their urban counterparts. Moreover, men showed a higher percentage of physical activity levels compared to women [12] and these latter reported that the social environment (e.g., guilt, family responsibility, social support) may significantly affect their physical activity level [13]. Therefore, investigations on the disparities between rural and urban context in physical activity are essential to promote the participation in physical activity, reduce medical costs and to adopt sustainable public health policies. Among the factors that promote participation in physical activity, volition in exercise, a psychological construct that is deemed essential to transform intention into concrete action [14] is one of the crucial factors to increase physical activity levels [15]. The concept of volition refers to an individual’s self-regulatory mental processes that are responsible for undertaking and maintaining a desirable behavior or action. Therefore, identifying specific volitional factors related to urban or rural contexts is fundamental to predict adherence to physical activity. To accurately predict exercise behavior, the Physical Exercise Volition Questionnaire (VEQ), that is highly specific to the exercise context, has been developed [14]. The VEQ proves to be a valuable tool as it directly assesses the availability to engagement in exercise and it is adapted to the active lifestyle domain, making it a suitable measurement tool for understanding and predicting exercise-related actions [14].

The aim of this study was to evaluate whether individuals that grew-up in rural and urban settings show different values in volition in exercise and physical activity levels considering sex differences. A secondary aim of our study was to explore the correlation between physical activity levels and volition in exercise in males and females that grew-up in different living settings.

## Materials and methods

### Participants

A total of 566 university students (373 females: BMI = 22.2 ± 3.3 kg/m^2^, 193 males: BMI = 23.9 ± 3.3 kg/m^2^) with an average age of 22.3 ± 2.6 years old were recruited. Inclusion criteria were participants currently enrolled in universities degree programs and aged between 18 and 35 years old.

Participation in the study was entirely voluntary, and it did not pose any risks or discomfort to the participants. Data privacy and confidentiality were rigorously ensured and participants signed a written informed consent. The study was reviewed and approved by the Centro di Ricerca e di Intervento Psicologico (CeRIP) ethics committee (University of Messina) before the study began (Approval Number 30152/2023).

### Experimental procedure

The data were collected via internet using the Google Form platform with a questionnaire developed by the authors between 3–30^th^ May 2023. The full survey is reported in S1 Appendix. Participants were recruited at the University “Magna Graecia” of Catanzaro while attending university lectures. The Google Form was also shared through social media channels as a 24-hour advertisement. Constant monitoring of access to the virtual platform was maintained throughout the whole experimental protocol. The questionnaire was divided in three different sections, and it required approximately 15 minutes to be completed. Specifically, the questionnaire was composed by:

1. Demographic Information (8 items): this section gathered participant information, including demographic data such as age, sex, and place where individuals lived until their eighteenth birthday. Based on this latter information rural or urban contexts were defined. Since at present no unique definition of rural and urban contexts exists [16] and the definition of rural and urban contexts may differ based on the nation of interest, to classify rural or urban contexts we referred to the information reported in the Italian national statistics institute [17]. Rural or urban context were defined based on the number of inhabitants and their population density (Rural: population density <300 inhabitants/km^2^; population: <5000 inhabitants; Urban: population density >300 inhabitants/km^2^; population: >5000 inhabitants).
2. Physical Activity Levels (16 items): The Global Physical Activity Questionnaire (G-PAQ) consisted of 16 items categorized into three domains: work, transportation, and recreational activities. The G-PAQ total score including all domains was used for the analysis [18].
3. Volition in Physical Exercise (18 items): Volition was measured using the Italian version of the Volition in Exercise Questionnaire [19]. It consisted of eighteen items scored on a rating scale ranging from zero ("it doesn’t match at all") to three ("exactly matches"). The VEQ-1 encompassed four volitional inhibition factors (VI) hindering an individual’s goal achievement and two volitional facilitation factors (VF) promoting goal attainment. The VI of the VEQ-1 included Reasons, Postponing Training, Unrelated Thoughts, and Approval from Others, while the VF consisted of Self-Confidence and Coping with Failure [19].

### Statistical analysis

A priori power analysis calculation (G*Power 3.1.9.2 software) showed that a total sample size of 504 participants and a medium effect size of 0.25 would provide a power of 0.8. Before further analysis, the normal distribution of the dependent variables was tested by applying the Kolmogorov-Smirnov test. This test showed that variables of interest (G-PAQ levels and VEQ-1 subscales) had skewed distributions. Therefore, Mann-Whitney U Test for independent samples was conducted to detect the differences between (a) females that grew-up in rural and urban context, (b) males that grew-up in rural and urban context as well as to investigate distinctions between females and males in the rural and urban context respectively. Moreover, to investigate the association between the abovementioned variables, Spearman correlation analysis was carried out separately for (c) rural females, (d) urban females, (e) rural males and (f) urban males. The level of significance was set at p < 0.05. Statistical analysis was carried out using IBM^®^SPSS software version 23.0 (SPSS Inc., Chicago, IL, USA).

## Results

Summary statistics of demographic information and the main variables (G-PAQ and VEQ-1 subscales) of the sample are reported in Tables 1 and 2 respectively.

**Table 1:** Demographic information of the sample. Age, Body mass, Height, Body mass index values are expressed as mean ± standard deviation.

**Variables**	**Females**	**Males**
**Age (years)**	22.2 ± 2.5	22.5 ± 2.8
**Body mass (kg)**	59.4 ± 9.7	75.5 ± 14.2
**Height (m)**	1.65 ± 0.44	1.77 ± 0.12
Body mass index (kg/m^2^)	22.2 ± 3.3	23.9 ± 3.6
**Education level**		
**Bachelor (n)**	196	89
**Master (n)**	57	42
**Single-cycle degree course (n)**	120	62
**Region 0–18 years**	**Living setting 0–18 years**	
	**Females**	**Males**
	**U/R**	**U/R**
**Basilicata (n/n)**	1/0	0/0
**Calabria (n/n)**	134/179	63/94
**Campania (n/n)**	8/6	1/4
**Emilia Romagna (n/n)**	1/0	1/0
**Lazio (n/n)**	7/10	7/10
**Lombardia (n/n)**	0/1	2/0
**Marche (n/n)**	0/1	0/1
**Puglia (n/n)**	2/3	5/2
**Sardegna (n/n)**	4/6	0/0
**Sicilia (n/n)**	4/3	2/1
**Toscana (n/n)**	0/1	0/0
**Trentino Alto Adige (n/n)**	1/0	0/0
**Umbria (n/n)**	1/0	0/0

n = number of participants; U = Urban; R = Rural

**Table 2:** Summary statistics of the main variables (G-PAQ and VEQ-1 subscales). Table’s values are expressed as mean ± standard deviation.

Variable	Females	Males
**G-PAQ (METs/wk)**	1939.3 ± 2339.6	3008.5 ± 2854.3
**Reasons**	3.2 ± 1.9	3.1 ± 2.0
**Postponing training**	3.6 ± 3.5	2.0 ± 2.7
**Unrelated thoughts**	2.6 ± 2.5	1.8 ± 1.9
**Approval from others**	2.7 ± 2.0	2.8 ± 1.9
**Self-Confidence**	5.2 ± 2.4	6.7 ± 2.3
**Coping with failure**	5.7 ± 2.0	6.3 ± 1.9

G-PAQ = Global Physical Activity Questionnaire; METs/wk = Metabolic Equivalents/week

Four-hundred sixteen students belonged to “medical study” field, 87 from “pharmacy and nutraceutical” field and 63 from “law, economics, and sociology” field. No significant differences between rural and urban females on G-PAQ levels and volition inhibitors and facilitators were found (p>0.05; Table 3). Moreover, no significant differences between rural and urban males on G-PAQ levels and volition inhibitors and facilitators were found (p>0.05; Table 3).

**Table 3:** Differences between rural and urban females and males. Results are reported as median (interquartile range).

Variables	Females *(n = 373)*		Males *(n = 193)*	
	R *(n = 164)*	U *(n = 209)*	R *(n = 97)*	U *(n = 96)*
**G-PAQ (METs/wk)**	1320.0 (2280.0)	1360.0 (2300.0)	2340.0 (2884.0)	2400.0 (3300.0)
**Reasons**	3.0 (3.0)	3.0 (3.0)	3.0 (3.0)	3.0 (3.0)
**Postponing training**	3.0 (6.0)	3.0 (4.0)	1.0 (3.0)	1.0 (3.5)
**Unrelated thoughts**	2.0 (4.0)	2.0 (4.0)	1.0 (3.0)	1.0 (3.0)
**Approval from others**	2.0 (3.0)	3.0 (3.0)	3.0 (2.8)	3.0 (3.0)
**Self-Confidence**	6.0 (3.0)	6.0 (4.0)	7.0 (4.0)	7.0 (4.0)
**Coping with failure**	6.0 (2.0)	6.0 (3.0)	6.0 (3.0)	6.0 (2.0)

R = Rural; U = Urban; G-PAQ = Global Physical Activity Questionnaire; METs/wk = Metabolic Equivalents/week

G-PAQ levels were significantly lower in rural females than rural males (p<0.01; Table 4). Concerning the volition inhibitors, postponing training and unrelated thoughts were significantly higher in rural females than rural males (p<0.01 and p<0.05 respectively; Table 4). Regarding volition facilitators self-confidence was significantly lower in rural females than rural males (p<0.01; Table 4). No significant differences on the other volition inhibitors (reasons, approval for others) and facilitators (coping with failure) were found (Table 4).

**Table 4:** Rural and urban sex differences. Results are reported as median (interquartile range).

Variables	Rural		Urban	
	F *(n = 164)*	M *(n = 97)*	F *(n = 209)*	M *(n = 96)*
**G-PAQ (METs-wk)**	1320.0 (2280.0) **	2340.0 (2884.0)	1360.0 (2300.0) **	2400.0 (3300.0)
**Reasons**	3.0 (3.0)	3.0 (3.0)	3.0 (3.0)	3.0 (3.0)
**Postponing training**	3.0 (6.0) **	1.0 (3.0)	3.0 (4.0) **	1.0 (3.5)
**Unrelated thoughts**	2.0 (4.0) *	1.0 (3.0)	2.0 (4.0) *	1.0 (3.0)
**Approval from others**	2.0 (3.0)	3.0 (2.8)	3.0 (3.0)	3.0 (3.0)
**Self-Confidence**	6.0 (3.0) **	7.0 (4.0)	6.0 (4.0) **	7.0 (4.0)
**Coping with failure**	6.0 (2.0)	6.0 (3.0)	6.0 (3.0) **	6.0 (2.0)

F = females; M = males; G-PAQ = Global Physical Activity Questionnaire; METs/wk = Metabolic Equivalents/week;

*p<0.05 vs M;

**p<0.01 vs M.

G-PAQ levels were significantly lower in urban females than urban males (p<0.01). The volition inhibitors, postponing training and unrelated thoughts were significantly higher in urban females than urban males (p<0.01 and p<0.05 respectively) (Table 4). The volition facilitators self-confidence and coping with failure were significantly lower in urban females than urban males (p<0.01) (Table 4). No significant differences on the other volition inhibitors (reasons, approval for others) were found (Table 4).

**Fig 1:**
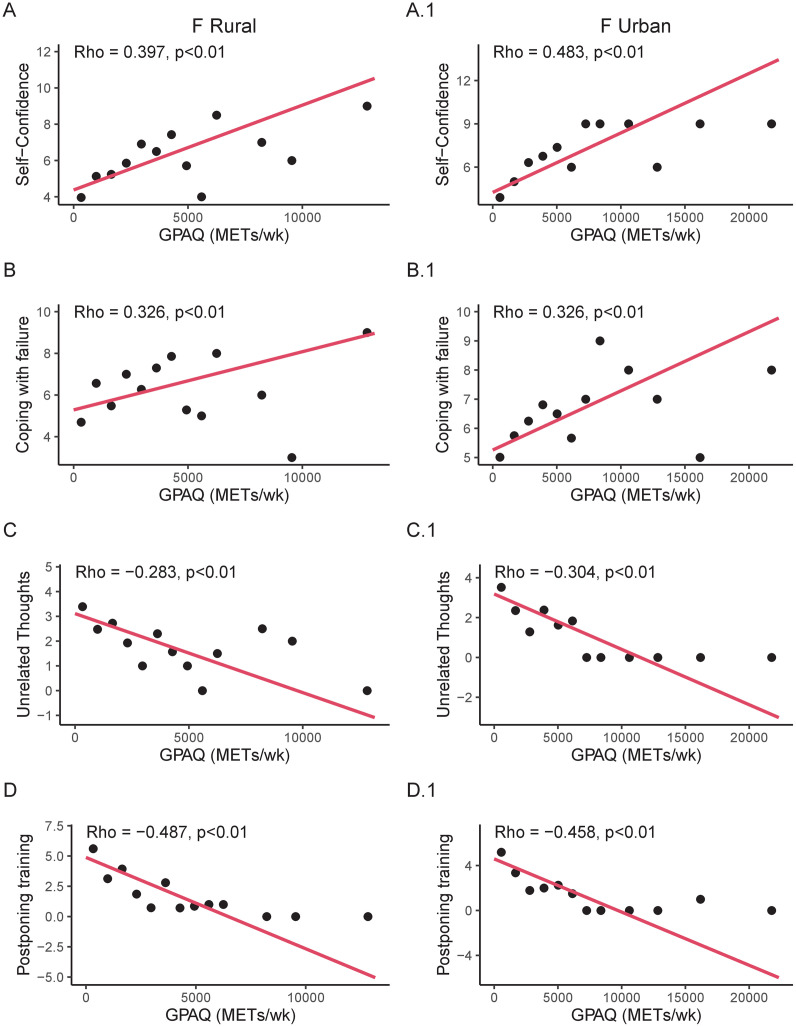
**Panel A and A.1.** Correlation analyses between Global Physical Activity Questionnaire (G-PAQ) and Self -Confidence in rural and urban females, respectively. **Panel B and B.1.** Correlation analyses between G-PAQ and Coping with failure in rural and urban females, respectively. **Panel C and C.1.** Correlation analyses between G-PAQ and Unrelated thoughts in rural and urban females, respectively. **Panel D and D.1.** Correlation analyses between G-PAQ and Postponing training in rural and urban females, respectively. All graphs are reported as binned scatterplots.

In rural females, G-PAQ levels positively correlated with self-confidence (Rho = 0.397, p<0.01) and coping with failure (Rho = 0.330, p<0.01). Moreover, G-PAQ levels negatively correlated with postponing training (Rho = -0.487, p<0.01) and unrelated thoughts (Rho = -0.283, p<0.01) (Fig 1). In urban females, G-PAQ levels positively correlated with self-confidence (Rho = 0.483, p<0.01) and coping with failure (Rho = 0.326, p<0.01). Moreover, G-PAQ levels negatively correlated with postponing training (Rho = -0.458, p<0.01) and unrelated thoughts (Rho = -0.304, p<0.01) (Fig 1).

**Fig 2:**
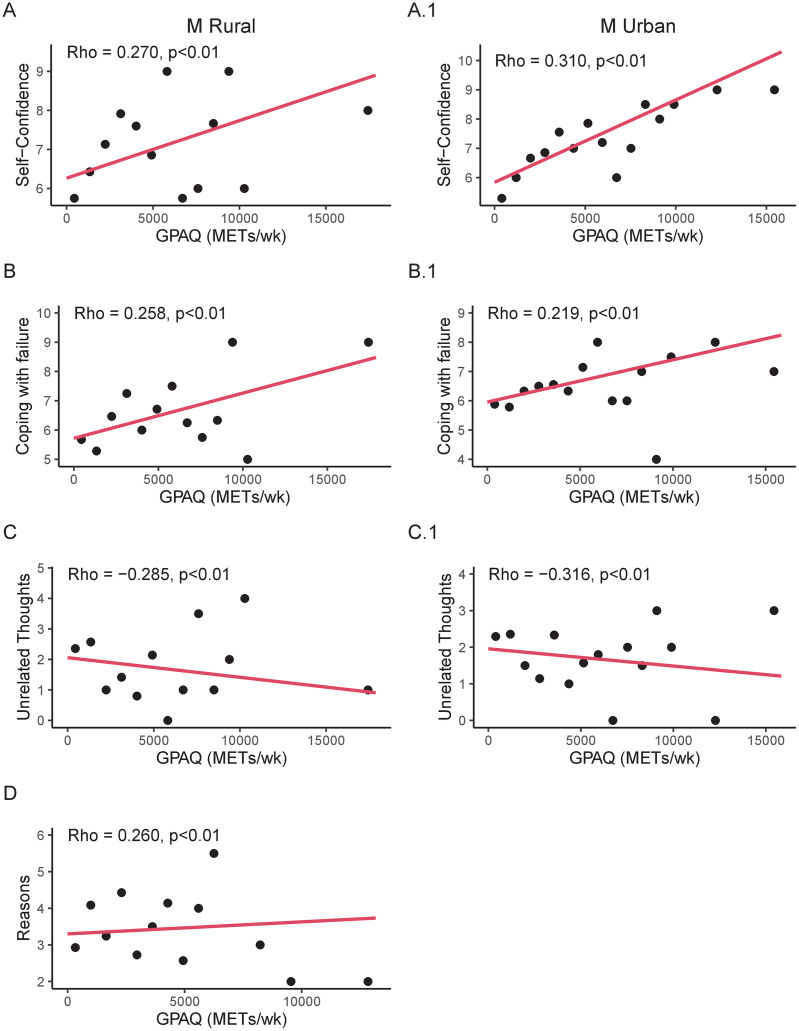
**Panel A and A.1.** Correlation analyses (reported as binned scatterplots) between Global Physical Activity Questionnaire (G-PAQ) and Self -Confidence in rural and urban males, respectively. **Panel B and B.1**. Correlation analyses between G-PAQ and Coping with failure in rural and urban males, respectively. **Panel C and C.1.** Correlation analyses between G-PAQ and Postponing training in rural and urban males, respectively. **Panel D.** Correlation analyses between G-PAQ and Reasons in urban males. All graphs are reported as binned scatterplots.

In rural males, G-PAQ levels positively correlated with self-confidence (Rho = 0.270, p<0.01) and coping with failure (Rho = 0.258, p<0.01; Fig 2). Moreover, G-PAQ levels negatively correlated with postponing training (Rho = -0.285, p<0.01; Fig 2).

In urban males, G-PAQ levels positively correlated with self-confidence (Rho = 0.310, p<0.01), coping with failure (Rho = 0.219, p<0.01) and reasons (Rho = 0.260, p<0.01; Fig 2). Moreover, G-PAQ levels negatively correlated with postponing training (Rho = -0.316, p<0.01; Fig 2).

## Discussion

We aimed to evaluate differences between males and females that grew-up in rural or urban living settings on physical activity levels and volition to exercise. As a secondary aim, we investigated the correlation between physical activity levels and volition factors considering also the context where individuals grew-up. Indeed, participation in different forms of physical activity (i.e., organized vs unorganized) might be influenced by environmental differences like rural and urban contexts [2, 12].

In the 21st century, physical activity levels have declined in both urban and rural contexts in Europe, but this decline has been more pronounced in rural environment [20]. Sedentary behavior may impact significantly on health and fitness status. Indeed, low levels of physical activity constitute risk factor for the onset of different of non-communicable diseases and may be associated with environment contextual-dependent circumstances [21]. Moreover, a recent study highlighted a ubiquitous change in perceived opportunities for physical activity. While urban residents once perceived more opportunities, this trend reversed in 2013, indicating changes in physical activity patterns between these two contexts [12].

Studies demonstrated that small-to-medium sized cities may be more advantageous for young people giving them a greater chance of achieving a higher level of physical activity [5–8]. Although large cities may offer young people better opportunity (e.g., well-designed, and equipped sports facilities), it appears that physical activity programs in large cities are highly structured and hampered by the lack of space and time in which individuals could participate [9, 10]. Interestingly, rural contexts offer favorable conditions for practicing physical activity especially unorganized physical activity, because of the availability of a wide range of stimuli [22]. At the opposite, previous research reported that the urban environment offers more opportunities for the practice of organized physical activity, but fewer possibilities for unorganized and outdoor physical activity compared to the rural environment [23]. Indeed, rural environments provide more space for physical activities and different type of sports and a safer environment to move around [24]. Smaller cities present a more favorable environment to practice sport compared to larger cities [25]. Therefore, rural and urban contexts offer unique advantages for the participation in physical activity. This diversity highlights the importance to consider different approaches to promote physical activity partecipation in these living settings. Our results showed no differences in physical activity levels between females and males that grew-up in rural or urban context until their eighteenth birthday. These similarities might be explained in the light of some considerations. Indeed, we considered the place where individuals lived until their 18th birthday while we assessed their current physical activity levels and volition. This aspect may account for the discrepancies with a previous study showing disparities in physical activity levels between rural and urban contexts [20]. However, our results are in line with a recent investigation showing comparable results between different living contexts in adolescents when assessed via questionnaires [16]. Moreover, the difficulties in the classification of rural and urban contexts added to the national and international demographic conformations prevent a comparison with previous studies on this topic.

The investigation of the psychological aspects of volition enabled us to gain insight into why some individuals engage in regular physical exercise or refrain from it [26]. Indeed, volition is one of the fundamental factors affecting physical activity levels [15]. By focusing on physical activity levels and volitional processes, we have shown that, in both contexts, females showed lower physical activity levels compared to males. However, in the rural context, females exhibited lower “self-confidence” compared to males, whereas in the urban context females showed lower “self-confidence” and “coping with failure” compared to males. Interestingly, the volition facilitator "self-confidence" emerged as an important predictor of the engagement in exercise activities [14]. Indeed, self-confidence is associated with the pre-actional phase, focusing on an individual’s belief in their own abilities to effectively confront challenges in adults [14]. Concerning the correlation between physical activity and volition factors, we showed that in females of both rural and urban context the GPAQ correlated positively with self-confidence and coping with failure and negatively with postponing training and unrelated thoughts. In rural males, physical activity levels correlated positively with self-confidence and coping with failure and negatively with postponing training. It has been shown that the volition inhibitor "Postponing training" impacts the shift from the pre-actional to the actional phase, marking the transformation of intention into concrete action. Therefore, this factor may represent a fundamental barrier to the engagement in regular physical activity. These results are in partial agreement with relevant literature on this topic [14] that focused on the relation between physical activity and volition factors. Unfortunately, this study neglected sex differences in the volition processes underpinning the participation in physical activity [14]. Our results showed that the volition inhibitor postponing training was higher in females of both living contexts compared to males. In a recent study [27], the lack of social support appears to be particularly relevant for women. Some researchers have highlighted the importance of social support for engaging women in physical activity, often linked to caring responsibilities, social resources, and easy access to sports facilities [28–33]. Reduced sex responsibilities (e.g., childcare, household duties) are associated with greater odds of meeting physical activity guidelines in rural women [34]. Therefore, targeted interventions to address sex-specific barriers may be effective in promoting physical activity participation in women [35]. In particular, social policies aiming at increasing social support in women may foster their participation in physical activity. As a side consideration, we did not investigate the availability of facilities in the two contexts. Previous studies reported that the presence of obstacles such as limited facilities, longer distances, insufficient public transport, and fewer pavements and cycling paths can limit both transport and leisure-time physical activities [36, 37]. It should be noted that the study presents some limitations. The self-reported questionnaire may have under or overestimated the amount of physical activity of the participants. The answer of our survey may be influenced by the intrinsic gender differences. Indeed, men may be more likely to be overconfident than women influencing the results of our survey [38].

Moreover, we did not collect information regarding the socio-economic status which is known to influence physical activity participation. Future studies should aim at understanding the evolution over time of the volitional processes underpinning physical activity participation on a larger sample considering different contexts.

## Conclusion

In conclusion, the place in where individuals grew-up (rural and urban contest) did not influence volitional processes and level of physical activity in both females and males. The correlations between physical activity levels and various volition factors were found to be consistent across rural and urban populations and between sexes, highlighting the role of psychological factors in physical activity participation. These findings suggest the importance of considering sex and psychological aspects to adopt sustainable public health policies when promoting physical activity during growth.

## Supporting information

S1 Appendix (PDF)

S1 File Minimal data set. (XLSX)
