# Supplementary material for: Influence of living settings on physical activity levels and volition in exercise in male and female university students
Source: PLoS One. 2024 Jul 18;19(7):e0304579. doi: 10.1371/journal.pone.0304579 (PMC11257310; doi:10.1371/journal.pone.0304579)
Supplement: S1 Appendix — (PDF) [file pone.0304579.s001.pdf]

Sustainability Research

Dear participant,

we kindly request your collaboration for the research project which aims at investigating how different living settings in which you grew-up influence our lifestyle, volition in exercise and physical activity levels in university students.

Study participation involves the administration of an online questionnaire that requires a total expected commitment of approximately 10 minutes. Participation is voluntary, free and does not entail any type of risk or discomfort.

To protect your privacy, we would like to assure you that your answers are anonymous and confidential. Data will only be used for statistical purposes in accordance with European Regulation 2016/679. Personal data will not be communicated and/or diffused to third parties without explicit consent; will not be subject to any full automated decision-making process, including profiling, according to art.22, paragraphs 1 and 4, of GDPR no.679/2016.

The head of research is Prof. Gian Pietro Emerenziani of the University "Magna Graecia" of Catanzaro.

For any information, you can send an email to: labsefes@unicz.it

If you agree to participate, please continue by filling out the questionnaire.

1. I confirm that I have read and agree with the information provided and am willing to participate in the study.

◯ I agree to participate.

◯ I do not agree to participate.

2. Since this is anonymous research, we do not have any data to contact you. If you would like to know the results of the questionnaires, please enter your email. If not, leave the box below blank.

---

3. 1. Indicate your biological sex (i.e. the sex assigned at birth).

◯ Female

◯ Male

4. 2. Indicate your age (in numbers):

---

5. 3. What course of study are you currently enrolled in? (e.g. Physical education, medicine, psychology, etc.)

6. 4. What is the level of your degree course?

◯ Bachelor's degree

◯ Master’s degree

◯ Single-cycle degree course

8. 6. In which city (or country) did you live until the age of 18?

---

9. 7. In which area of the city (or country) did you live until you were 18 years old?

◯ Center

◯ Suburbs

14. 12. How much is your currently weight? (Specify in kilograms, using a comma for decimals, e.g. 73,8 kg).

---

15. 13. How tall are you? (in centimeters)

GLOBAL PHYSICAL ACTIVITY QUESTIONNAIRE (GPAQ)

INSTRUCTIONS FOR COMPLETION

In the next questions, we investigate how much time, in a normal week, you dedicate to different physical activities. Please answer these questions even if you don't consider yourself to be a physically active person.

21. 1) In a typical week, on how many days do you walk or bicycle for at least 10 minutes continuously to get to and from places?

Mark only one oval.

◯ Never (go to question No.3)

◯ 1

◯ 2

◯ 3

◯ 4

◯ 5

◯ 6

◯ 7

22. 2) How much time do you spend walking or bicycling for travel on a typical day? (Indicate the minutes, e.g. 1,5h=90min)

____

23. 3) Do you do any vigorous-intensity sports, fitness or recreational (leisure) activities that cause large increases in breathing or heart rate like [running or football] for at least 10 minutes continuously?

Please Note: Strenuous physical activities cause breathing to be much stronger than normal. Examples of strenuous physical activity:

- Soccer
- Rugby
- Tennis
- High impact aerobic exercise
- In water aerobic exercise
- Dance
- Fast swimming

Mark only one oval.

◯ Yes

◯ No (go to question No.6)

24. 4) In a typical week, on how many days do you do vigorous - intensity sports, fitness or recreational (leisure) activities?

Mark only one oval.

◯ 1

◯ 2

◯ 3

◯ 4

◯ 5

◯ 6

◯ 7

25. 5) How much time do you spend doing vigorous-intensity sports, fitness or recreational activities on a typical day? (Indicate the minutes, e.g. 1,5h=90min)

____

26. 6) Do you do any moderate-intensity sports, fitness or recreational (leisure) activities that cause a small increase in breathing or heart rate such as brisk walking, [cycling, swimming, volleyball] for at least 10 minutes continuously?

Please Note: Moderate physical activities cause breathing to be a little stronger than normal. Examples of intense physical activity:

- Cycling
- Jogging
- Dance
- Horse riding
- Tai chi
- Yoga
- Pilates
- Low impact aerobic exercise
- Cricket

Mark only one oval.

◯ Yes

◯ No (go to question No.9)

27. 7) In a typical week, on how many days do you do moderate-intensity sports, fitness or recreational (leisure) activities?

Mark only one oval.

◯ 1

◯ 2

◯ 3

◯ 4

◯ 5

◯ 6

◯ 7

28. 8) How much time do you spend doing moderate-intensity sports, fitness or recreational (leisure) activities on a typical day? (Indicate the minutes, e.g. 1,5h=90min)

____

29. 9) How much time do you usually spend sitting or reclining on a typical day? (Indicate hours)

The following question is about sitting or reclining at work, at home, getting to and from places, or with friends including time spent sitting at a desk, sitting with friends, traveling in car, bus, train, reading, playing cards or watching television, but do not include time spent sleeping.

____

VOLITION IN EXERCISE QUESTIONNAIRE

INSTRUCTIONS FOR COMPLETION

Score each affirmation.

0 (does not correspond at all); 3 (corresponds exactly)

31. 1. I feel I have to meet others' expectations during my exercise activity.

0 1 2 3

Does not ◯ ◯ ◯ ◯ Corresponds exactly

32. 2. I only begin my exercise activity when I am pressured to it.

0 1 2 3

Does not ◯ ◯ ◯ ◯ Corresponds exactly

33. 3. During my exercise activity, I often find it difficult to concentrate because I start thinking of things not connected to the activity itself.

0 1 2 3

Does not ◯ ◯ ◯ ◯ Corresponds exactly

34. 4. I believe in my own ability to do well in my exercise activity.

0 1 2 3

Does not ◯ ◯ ◯ ◯ Corresponds exactly

35. 5. I am convinced that I am able to carry out strenuous exercise activities.

0 1 2 3

Does not ◯ ◯ ◯ ◯ Corresponds exactly

36. 6. During my exercise activity I often focus on my reason for participating.

0 1 2 3

Does not ◯ ◯ ◯ ◯ Corresponds exactly

37. 7. When I make a mistake during my exercise activity, I quickly move on.

	0	1	2	3	
Does not	◯	◯	◯	◯	Corresponds exactly

38. 8. I adapt to others during my exercise activity.

	0	1	2	3	
Does not	◯	◯	◯	◯	Corresponds exactly

39. 9. I wait to embark on my exercise activity until there is no way around it.

	0	1	2	3	
Does not	◯	◯	◯	◯	Corresponds exactly

40. 10. During my exercise activity, I am disturbed by thoughts that are not related to the activity itself.

	0	1	2	3	
Does not	◯	◯	◯	◯	Corresponds exactly

41. 11. I believe that my will is strong enough to carry out strenuous exercise activities.

	0	1	2	3	
Does not	◯	◯	◯	◯	Corresponds exactly

42. 12. During my exercise activity, I find it difficult to concentrate because my thoughts drift to other things than the activity itself.

	0	1	2	3	
Does not	◯	◯	◯	◯	Corresponds exactly

43. 13. If I make a mistake during my exercise activity, I am quick to improve my effort.

	0	1	2	3	
Does not	◯	◯	◯	◯	Corresponds exactly

44. 14. I am afraid of what others think of me if I do not perform as expected in my exercise activity.

	0	1	2	3

Does not ◯ ◯ ◯ ◯ Corresponds exactly

45. 15. I often experience that I really need to pull myself together to participate in my exercise activity.

	0	1	2	3	
Does not	◯	◯	◯	◯	Corresponds exactly

46. 16. I think a lot about my reason for participating in my exercise activity.

	0	1	2	3	
Does not	◯	◯	◯	◯	Corresponds exactly

47. 17. Even though my exercise activity is not going well, it is easy for me to get back on track.

	0	1	2	3	
Does not	◯	◯	◯	◯	Corresponds exactly

48. 18. I wait until the last minute before I begin my exercise activity.

	0	1	2	3	
Does not	◯	◯	◯	◯	Corresponds exactly
